# Susceptibility Profile and Multiple Antibiotics Resistance of *Escherichia coli*, *Klebsiella* spp., and *Enterococci* from Small-Scale Cattle Farms in Tennessee

**DOI:** 10.3390/antibiotics15020217

**Published:** 2026-02-17

**Authors:** Goodness Olakanmi, Maureen Nzomo, Bharat Pokharel, Abdullah Mafiz, Agnes Kilonzo-Nthenge

**Affiliations:** Department of Food and Animal Sciences, Tennessee State University, 3500 John A. Merritt Boulevard, Nashville, TN 37209, USA; golakanm@my.tnstate.edu (G.O.); mo3nana@gmail.com (M.N.); bpokhare@tnstate.edu (B.P.); abdullah.mafiz@gmail.com (A.M.)

**Keywords:** antimicrobial resistance, *Escherichia coli*, *Klebsiella*, *Enterococcus*, small-scale cattle farms, practices

## Abstract

**Background/Objectives**: Antimicrobial resistance in food–animal environments threatens sustainable production and public health, yet small farms remain poorly characterized as potential reservoirs of antimicrobial resistant bacteria. To address this, we investigated the prevalence and antimicrobial resistance profiles of *Escherichia coli*, *Klebsiella* spp., and *Enterococcus* spp. from small-scale cattle farms in Tennessee, USA. **Methods**: Over one year, 153 environmental samples (soil, manure, water) were collected from 17 farms. Target bacteria were isolated and confirmed using selective agar, biochemical tests, and PCR, and tested against 12 antibiotics using the Kirby–Bauer disk diffusion test. Multiple Antibiotic Resistance Index (MARI) and multidrug resistance (MDR) profiles were summarized. A complementary farmer survey of 26 farmers captured veterinary access, antibiotic use, manure handling, record keeping, and awareness of antimicrobial resistance. **Results**: Prevalence was highest for *Enterococcus* spp. (41.8%), followed by *E. coli* (23.5%) and *Klebsiella* spp. (12.4%). Seasonal variation was significant for *E. coli* and *Enterococcus* (*p* < 0.05). Winter manure yielded highest detection of *E. coli* (55.6%) and *Enterococcus* (53.8%), whereas *Klebsiella* peaked in Fall soil (19.1%). Resistance patterns varied across species, with *Enterococcus* showing consistent resistance to all three. *E. coli* frequently resisted erythromycin, ampicillin, and azithromycin; and *Klebsiella* commonly resisted erythromycin, ampicillin, and cefotaxime, though some of these reflect intrinsic resistance rather than acquired clinical resistance. MARI values were 0.92 in manure and soil, identifying them as high-risk reservoirs. We identified 29 distinct MDR pattern. Bipartite network visualization highlighted “resistance hubs” around erythromycin, ampicillin, and vancomycin, particularly in *Enterococcus*. In our study, 76.9% of farmers consulted veterinarians before antibiotic use, 57.7% kept written antibiotic records, and 65.4% were aware of AMR as a public health issue. Small-scale cattle farms are potential reservoirs of multidrug resistant commensal bacteria. **Conclusions**: These findings provide an evidence-based foundation to guide targeted antimicrobial stewardship and promote sustainable management practices in small-scale food animal farms.

## 1. Introduction

Antimicrobial resistance (AMR) is a growing global health crisis, with projections estimating up to 10 million annual deaths by 2050 if current trends continue [1]. Resistant bacteria emerge and spread through multiple pathways, including direct contact with livestock, consumption of contaminated food, and environmental exposure via soil, water, and manure [2,3]. Food–animal production environments act as important reservoirs where resistant bacteria can persist, exchange resistance determinants, and disseminate across ecosystems [4]. The mobility of these determinants through runoff, manure application, and hydrological networks further amplifies risk [5].

Small farms play a critical role in local food security and rural economies yet often operate with limited access to AMR surveillance systems, veterinary oversight, and structured stewardship programs [6,7]. Most AMR research has focused on large, intensive operations; however, smaller farms particularly in regions with diverse management practices and fewer resources remain under-documented in the literature, despite growing evidence that informal veterinary access, variable biosecurity, and inconsistent antibiotic use may contribute to resistance emergence in these settings [8,9]. These structural factors, which are also commonly reported in low- and middle-income countries (LMICs), show the need to understand AMR risks not only across nations but also within under-regulated production systems globally. Recent LMIC studies have highlighted how misuse of antibiotics, weak biosecurity, and environmental dissemination sustain AMR within small-scale farm environments even in high income countries [10,11]. Understanding AMR dynamics in these settings is essential to developing context-specific mitigation strategies and advancing sustainable food production.

Among pathogens of concern, *Escherichia coli* (*E. coli*), *Klebsiella* spp., and *Enterococcus* spp. are recognized by the U.S. Centers for Disease Control and Prevention (CDC) for their role in both environmental persistence and clinical infections [12]. *Klebsiella pneumoniae*, in particular, is part of the ESKAPE group of pathogens known for high-risk hospital-acquired infections transmission and multidrug resistance, while *E. coli* and *Enterococcus* spp. are designated as high-priority organisms on the World Health Organization’s global list of AMR threats [13,14,15,16]. Although often commensal, these bacteria serve as important reservoirs of resistance genes and are associated with a range of opportunistic infections in both human and veterinary medicine [13]. In broader context beyond the scope of this study, Extended-spectrum β-lactamase (ESBL)-producing *E. coli* and carbapenem-resistant *Klebsiella* have been reported in agricultural systems worldwide [14,15,16], while *Enterococcus faecalis* and *E. faecium* serve as key reservoirs of vancomycin resistance [17,18,19]. Prolonged antimicrobial use in production environments continues to drive multidrug resistance, complicating treatment outcomes in both veterinary and human medicine [20,21].

This study addresses critical gaps in AMR within small-scale cattle farms in Tennessee, USA. Specifically, it evaluates the prevalence and susceptibility profiles of *E. coli*, *Klebsiella* spp., and *Enterococcus* spp. in soil, manure, and water across seasons, and infers resistance intensity using the Multiple Antibiotic Resistance Index (MARI). In addition, it explores farmer management practices related to antibiotic use and manure handling. These data provide locally relevant evidence that identifies high-risk environmental reservoirs of multidrug-resistant commensal bacteria and supports targeted antimicrobial stewardship strategies for small-scale food systems.

## 2. Results

### 2.1. Prevalence of Bacterial Isolates

#### 2.1.1. *Enterococcus* spp.

**Figure 1:**
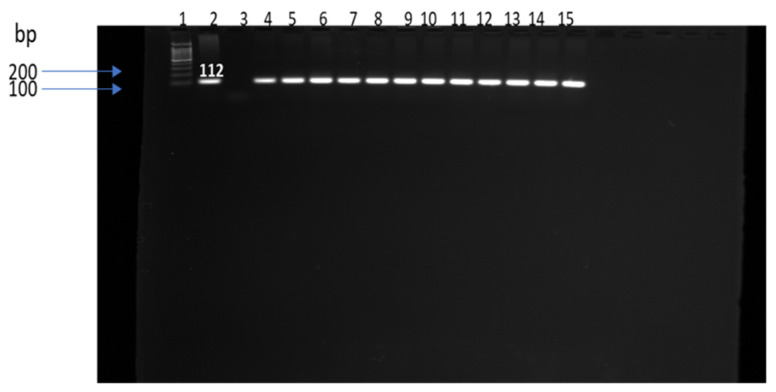
PCR amplification of *tuf* gene in *Enterococcus* spp. Lane 1:100 bp DNA marker, lane 2: positive control (*Enterococcus faecium* ATCC 19434, lane 3: negative control (water), lanes 4 through 15: DNA samples, bp: base pairs.

Out of 153 samples analyzed, *Enterococcus* spp. were recovered from 64 (41.8%) when tested by API Strep (bioMérieux). Manure yielded the highest detection rate (68.5%, 37/54), followed by water (44.4%, 16/36) and soil (17.5%, 11/63). Seasonal variation was significant (*p* < 0.05). In winter, manure (53.8%, 7/13) and soil (41.7%, 5/12) showed the highest recovery, while no isolates were detected in water. In spring, prevalence was comparable between manure (23.8%, 10/42) and soil (23.8%, 10/42). In summer, manure maintained the highest recovery (30.9%, 13/42), compared with soil (11.9%, 5/42) and water (5.7%, 2/35). During fall, water exhibited the highest prevalence (25.7%, 9/35), compared with manure (13.7%, 7/51). PCR amplification of the *tuf* gene confirmed 30 representative isolates. Figure 1 presents a gel image showing 12 of the 30 confirmed isolates.

#### 2.1.2. *E. coli*

**Figure 2:**
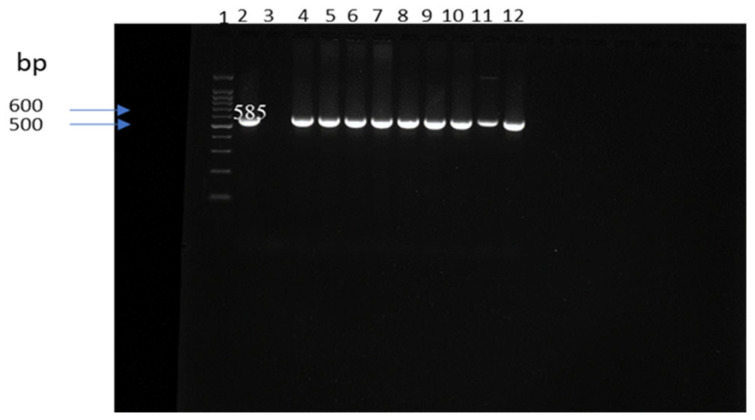
PCR amplification of the 16S rRNA gene in *E. coli*. Lane 1:100 bp ladder, lane 2: positive control (*E. coli* ATCC 11775), lane 3: negative control (water), lanes 4–12: DNA samples, bp: base pairs.

A total of 36 isolates (23.5%) were identified as *E. coli* by API 20E and all the isolates were confirmed by PCR amplification of the 16S rRNA gene. Figure 2 shows a representative subset of 9 confirmed isolates. Detection was intense in manure samples across all seasons except spring. In winter, manure showed the highest prevalence (55.6%, 5/9), compared with soil (18.2%, 2/11) and water (8.3%, 1/12) (*p* < 0.05). In fall, *E. coli* was only recovered from manure (7/35; 20%). In spring, the prevalence was 6.7% (2/30) in manure and 14.3% (3/21) in soil, with no isolates from water. In summer, *E. coli* prevalence was 23.8% (10/42) in manure, 4.8% (2/42) in soil, and 11.8% (4/34) in water.

#### 2.1.3. *Klebsiella* spp.

**Figure 3:**
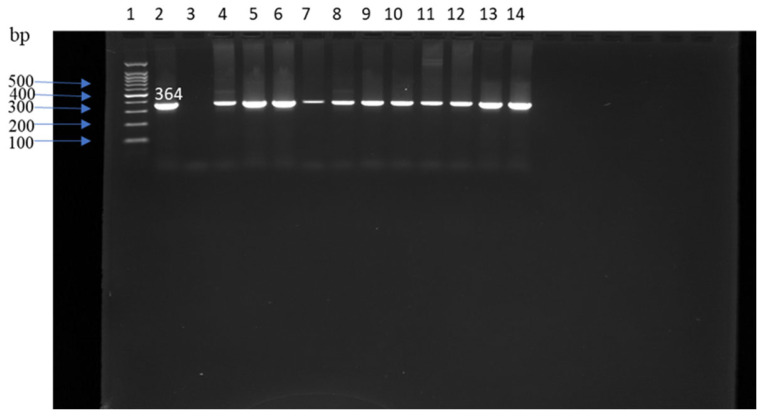
PCR amplification of the *mdh* gene in *Klebsiella* spp. Lane 1:100 bp ladder, lane 2: positive control (*Klebsiella Pneumoniae* ATCC 700603), lane 3: negative control (water), lanes 4 through 14: DNA samples, bp: base pairs.

*Klebsiella* spp. was the least prevalent group, detected in 19 isolates (12.4%) by API 20E. PCR targeting the *mdh* gene confirmed 11 isolates as *K. pneumoniae* (Figure 3). The highest prevalence occurred in fall soil samples (19.1%, 9/47), followed by spring manure (9.1%, 2/22), winter water (8.3%, 1/12), summer water (7.5%, 3/30), fall water (6.7%, 1/15), spring soil (4.0%, 1/250), fall manure (2.7%, 1/37) and summer soil (2.4%, 1/42). No *Klebsiella* spp. were recovered from winter manure or soil, summer manure, or spring water. Seasonal differences were not statistically significant (*p* > 0.05).

**Figure 4:**
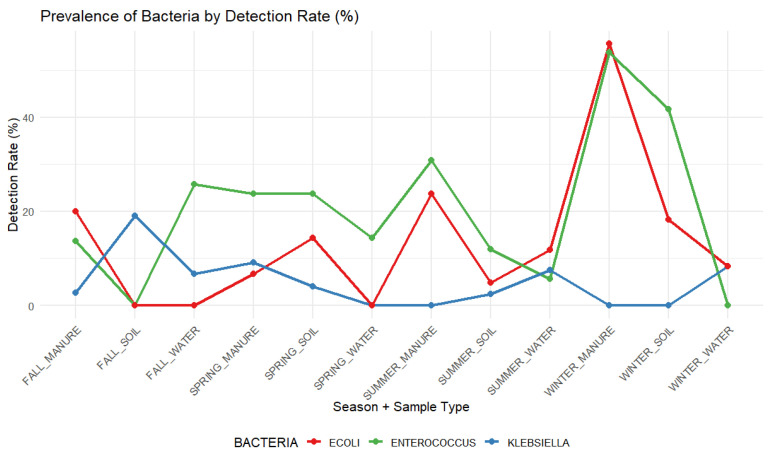
Seasonal prevalence of *E. coli*, *Enterococcus* spp., and *Klebsiella* spp. from environmental samples based on detection rate (%). Prevalence is shown by season and sample type (manure, soil and water), highlighting variations in bacterial detection across environmental sources and time.

Figure 4 illustrates bacteria prevalence trends by detection rate percentage. *Enterococcus* spp. exhibited the highest average detection rate overall, with peaks in winter manure (53.8%) and winter soil (41.7%). *E. coli* detection was the highest in winter manure (55.6%) and summer manure (23.8%). *Klebsiella* spp. showed lower detection rates overall, with the highest prevalence observed in fall soil (19.1%).

### 2.2. Antibiotic Susceptibility Profiles

**Figure 5:**
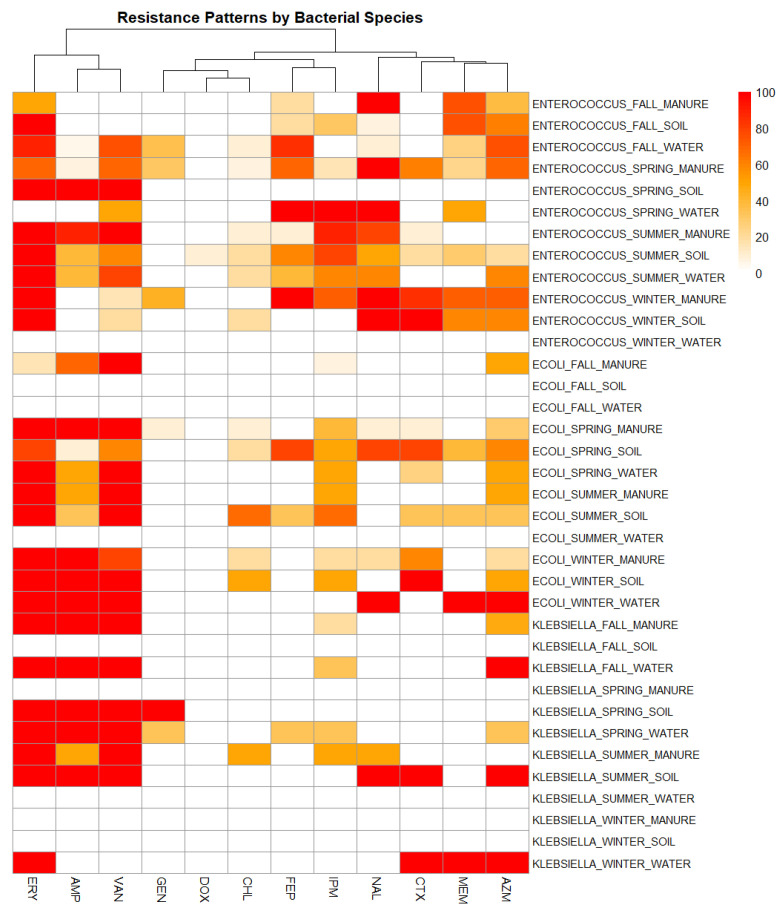
Heatmap showing antimicrobial resistance profiles of *Enterococcus* spp., *E. coli*, and *Klebsiella* spp. across sample types (manure, soil, water) and seasons (fall, spring, summer, winter). Resistance percentages are color-coded from 0% (white) to 100% (red). Antibiotics: erythromycin (ERY), ampicillin (AMP), vancomycin (VAN), gentamicin (GEN), doxycycline (DOX), chloramphenicol (CHL), cefepime (FEP), imipenem (IPM), nalidixic acid (NAL), cefotaxime (CTX), meropenem (MEM), and azithromycin (AZM).

Antimicrobial susceptibility testing against 12 antibiotics revealed clear interspecies and matrix-specific variation among the isolates as shown in Figure 5.

*Enterococcus* spp. displayed the broadest resistance spectrum. ERY resistance was consistently high, reaching 100% in all summer and winter manure and soil samples, and in fall soil, spring soil and summer water. NAL resistance peaked at 100% in fall and winter manure, winter soil, and all spring samples except soil. AMP resistance was the highest in spring soil (100%), summer manure (90%) and 40% in both summer soil and water. VAN resistance reached 100% in summer manure and spring soil, with notable levels in fall water (75%), in summer soil and water (60% and 80%), and in spring manure (69.2%). Carbapenem resistance was variable: IPM resistance peaked at 100% in spring water and 90% in summer manure; MEM was highest in fall manure and soil (75% each) and in winter manure (71.4%). CTX resistance reached 100% in winter soil and 85.7% in winter manure, with lower values in other seasons and matrices. FEP resistance peaked at 100% in winter manure and spring water, and 85% in fall water. In contrast, DOX, CHL and GEN showed consistently low resistance, with most values at or near 0% across sample types and seasons.

*E. coli* showed strong resistance to ERY, AMP and VAN. ERY resistance reached 100% in all winter samples, summer manure and soil, and spring manure and water. AMP resistance was 100% in all winter samples and spring manure. VAN resistance was consistently high in manure from all seasons with the lowest being 80% in winter. Complete VAN resistance (100%) was observed in summer and winter soil, as well as in winter and spring water. NAL resistance was generally low, except in winter water (100%) and spring soil (80%). CTX resistance peaked at 100% in winter soil, with moderate levels in spring soil (80%) and winter manure (60%). AZM resistance was the highest in winter water (100%) and spring soil (60%). MEM resistance was observed in the single isolate recovered in winter water and was absent in most other samples. IPM resistance varied, peaking in summer soil (66.7%), followed by 50% resistance in spring soil and water, summer manure, and winter soil. FEP resistance was mostly absent, with the highest value of 80% in spring soil, and 33.3% in summer soil. CHL resistance was also low overall, peaking at 66.7% in summer soil. GEN resistance was rare, with only 10% in spring manure. DOX resistance was absent across all isolates.

*Klebsiella* spp., though isolated less frequently, demonstrated distinct peaks of resistance. Soil and water isolates in spring, fall manure and water, and summer soil showed 100% resistance to ERY, AMP, and VAN. CTX resistance reached 100% in winter water and summer soil samples, indicating that on-farm drinking water can also harbor resistant *Klebsiella*. GEN resistance was uncommon but reached 100% in spring soil isolates. In contrast to *E. coli* and *Enterococcus*, *Klebsiella* isolates showed stronger seasonal contrasts, with several winter manure and soil samples showing no detectable resistance.

Overall, manure and soil were the dominant reservoirs of resistance, but occasional high values in farm water samples (e.g., ERY, AMP, VAN and AZM 100% resistance in fall *Klebsiella*) suggest that water within farm systems also contributes to persistence of resistant bacteria. These species- and matrix-specific profiles provide the basis for examining how resistance traits converge within individual isolates, which is explored in the following section on multidrug resistance diversity.

### 2.3. Resistance Pattern Diversity

**Figure 6:**
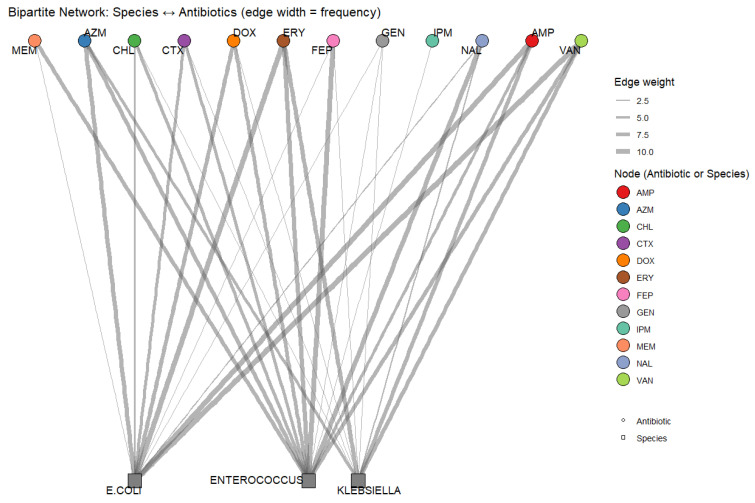
Bipartite network of bacterial resistance patterns. Species (*E. coli*, *Klebsiella* spp., *Enterococcus* spp.; squares) are linked to antibiotics (circles), with edge widths representing the frequency of observed resistance.

Across all isolates, 29 distinct multidrug resistance (MDR) profiles were identified (Supplementary Table S1). MDR was defined in accordance with Magiorakos criteria as acquired resistance to at least one agent in three or more antimicrobial categories [22]. The complexity of these combinations is summarized in the bipartite resistance network (Figure 6).

*Enterococcus* spp. exhibited the most diverse and extensive multidrug resistance (MDR) profiles. In spring soil and summer manure, isolates carried resistance to 11 antibiotics including ERY, NAL, AMP, MEM, FEP, VAN, CHL, AZM, CTX in both matrices. Other *Enterococcus* isolates from winter manure and soil, spring manure and water and summer soil demonstrated resistance 8–10 resistance combinations, suggesting a capacity for maintaining high-level MDR irrespective of seasonal or sample-type context.

*E. coli* displayed moderately complex resistance patterns. Several manure (summer and winter) and soil (spring, winter) isolates carried resistance to 8–9 antibiotics, frequently involving combinations of ERY, AMP, AZM, CHL, DOX, and CTX indicating common resistance themes. In contrast, water isolates generally showed narrower resistance ranges, rarely exceeding six antibiotics, reflecting a broader spread of the MDR complexity across this species.

*Klebsiella* spp. contributed fewer MDR profiles overall but showed notable complexity in certain matrices. The most extensive resistance pattern (seven antibiotics) was detected in a summer water isolate, which included ERY, AMP, FEP, VAN, GEN, DOX, and AZM. Spring manure and soil isolates also carried six-drug resistance profiles, though involving different combinations, such as CTX, CHL, or DOX. Fall samples consistently carried 4–5-drug profiles across soil, manure, and water, dominated by ERY, AMP, VAN, and AZM. In contrast, no *Klebsiella* isolates from winter manure or soil exhibited any resistance, reinforcing its lower prevalence and more seasonally constrained MDR contribution.

The bipartite network highlights both shared and species-specific resistance hubs (Figure 6). Antibiotics such as ERY, AMP, VAN, and AZM emerged as central nodes linking all three bacterial groups suggesting common selective pressures across farm environments. In contrast, antibiotics like MEM, CHL, NAL and DOX were more sparsely connected, predominantly associated with *Enterococcus* and subsets of *E. coli* and *Klebsiella* isolates. Collectively, while *Enterococcus* isolates carried the most complex MDR signatures, all three species contributed to the overall diversity of resistance combinations. The presence of shared resistance hubs signifies overlapping ecological roles and highlights antibiotic classes that may act as cross-species drivers of resistance persistence in small farm environments. While resistance hubs were visually apparent, we acknowledge that these inferences are based on phenotypic data alone. The absence of genotypic confirmation of resistance mechanisms represents a limitation of this study.

### 2.4. Multiple Antibiotic Resistance Index (MARI) Patterns

**Figure 7:**
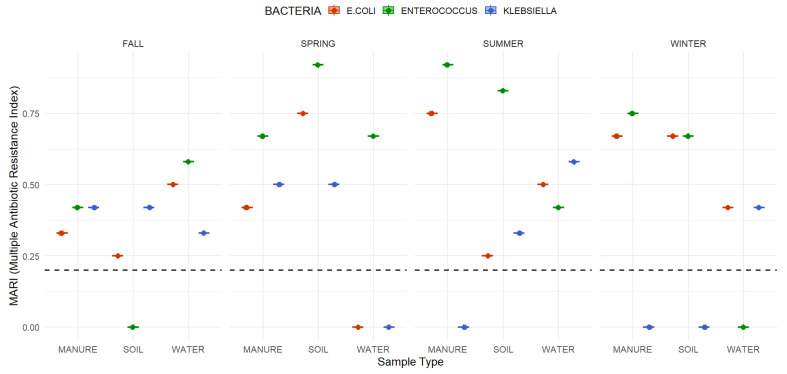
Multiple Antibiotic Resistance Index (MARI) values by bacterial species (*E. coli*, *Enterococcus* spp., *Klebsiella* spp.), season, and sample type (manure, soil, water). Dashed horizontal line represents the high-risk threshold (MARI = 0.2). Points represent average MARI values per species, sample type, and season.

MARI values ranged from 0.00 to 0.92 across bacterial species, sample types, and seasons (Figure 7 and Supplementary Table S1). *Enterococcus* spp. exhibited the highest indices, with values up to 0.92 in spring soil and summer manure. *E. coli* showed moderate MARI values, peaking at 0.75 in spring soil and summer manure, and values of ≥0.67 in winter soil and manure. In contrast, *Klebsiella* spp. generally displayed lower indices, with a maximum of 0.58 in summer water.

Most of the manure and soil isolates exceeded the 0.2 threshold, indicating environments with high antibiotic exposure. Water samples were more variable, with most values lower but occasional elevations such as 0.58 for *Enterococcus* in fall water. Overall, *Enterococcus* consistently carried the greatest resistance intensity, while *E. coli* and *Klebsiella* also maintained indices above the risk threshold in several seasons.

### 2.5. Adoption of Best Management Practices (BMPs)

Twenty-six Tennessee farmers completed the BMP survey. Their demographic characteristics and farm features are summarized in Table 1. Most respondents were male (92.3%), and the largest age group was 65 years and older (57.7%). The majority of participants (42.3%) had a high school diploma, while 15.4% held a Bachelor’s degree and 23.1% had a Master’s degree. Regarding antibiotic use, 69.2% of farmers reported using antibiotics to treat sick animals only, while 23.2% used them for both treatment and disease prevention. Written antibiotic records were kept by 57.7%, and 76.9% consulted a veterinarian before administration. Antibiotics were always used as recommended by all farmers.

Awareness of AMR as a public health issue was reported by 65.4% of respondents. Training in BMPs was received by 84.6%, and all sought guidance from extension agents. Manure management practices was largely stockpiling (100%) which may contribute to the persistence of resistance traits in soil and manure, as reflected in the elevated MARI values observed in these matrices. Dead animal disposal methods were always through dead animal service (100%). These patterns indicate relatively high engagement with veterinary services and awareness of AMR among survey farmers, though opportunities remain to improve record keeping and diversify manure management strategies.

## 3. Discussion

The seasonal and matrix variation in *Enterococcus*, *E. coli* and *Klebsiella* prevalence observed in this study suggests that environmental conditions influence bacterial persistence. *Enterococcus* was the most frequently detected bacterium, with the highest recovery in manure and notable peaks in winter manure and soil. This is consistent with reports identifying it as a robust environmental colonizer in livestock systems [23,24]. Recovery occurred across manure, soil and water, with manure showing consistently high detection rates, especially in winter. However, in fall, water exhibited the highest prevalence, highlighting seasonal and matrix-specific variation in *Enterococcus* persistence. This pattern aligns with its ability to persist under diverse environmental stressors and supports its use as an indicator of fecal contamination in agricultural environments [25]. Management practices such as manure handling, controlled grazing and water protection are therefore important for limiting *Enterococcus*, *E. coli* and *Klebsiella* persistence within farm systems.

Detection of *E. coli* across matrices highlights pathways of fecal contamination and suggests that unmanaged waste streams may contribute to sustained AMR transmission risk, particularly during wetter months when runoff potential is higher [26]. Higher prevalence in manure across seasons suggests this matrix is the principal source from which *E. coli* can move into soil and water. Previous work has shown that untreated manure can sustain *E. coli* and facilitate its transfer to soil, crops, and surface water [27,28,29]. Seasonal practices, such as manure application or grazing during wet conditions, can increase runoff and microbial dissemination [30,31], whereas carefully timed applications and grazing rotations may help reduce spread [32,33].

*Klebsiella* spp. was the least frequently detected group, consistent with its generally lower prevalence compared with other Enterobacteriaceae [34,35]. Several isolates were confirmed as *K. pneumoniae*, an opportunistic pathogen of clinical concern. Although seasonal differences were not statistically significant, detection across soil, water and manure indicates that *Klebsiella* can persist in farm environments and contribute to environmental reservoirs of resistance. Given its association with extended spectrum beta lactamase (ESBL) production, continued monitoring is warranted even at low prevalence [36,37].

The antimicrobial susceptibility profiles revealed distinct but overlapping resistance patterns among the three genera. *Enterococcus* showed the broadest and most intense resistance spectrum. High resistance to ERY, AMP and VAN is consistent with its role as a dominant environmental reservoir of AMR in farm settings [38,39]. The detection of carbapenem resistance in MEM and IPM in some *Enterococcus* isolates despite restricted agricultural use suggests a potential spillover from clinical or environmental sources. *E. coli* displayed moderately complex resistance patterns, particularly in manure and soil where there were peak rates of resistance to ERY, AMP and VAN. These may reflect sustained selective pressure in matrices where antibiotic residues or co-selective factors may persist. Although *Klebsiella* contributed fewer isolates, it showed marked resistance peaks in specific matrices. Notably, 100% resistance to CTX in winter water and summer soil isolates is concerning for potential ESBL production [40,41]. In addition, complete resistance to ERY and AZM suggests overlapping selective pressures from macrolide use or co-resistance mechanisms. Other antibiotics including AMP and VAN also exhibited complete resistance in select matrices, although not uniform in all species or seasons may reflect additional selective pressures in farm environments.

Resistance trends also reflected clear environmental and seasonal influences. Although IPM, MEM and GEN resistance was rare, their sporadic presence in both *Enterococcus* and *Klebsiella* warrants attention and the need for continued surveillance to track potential emergence from non-agricultural sources [37,42,43,44]. Resistance tended to peak in cooler, wetter periods, especially in winter and spring where manure and soil matrices consistently harbored multi-resistant isolates. In contrast, resistance appeared more variable or reduced during summer, though isolated peaks highlight the complexity of these dynamics. These patterns likely reflect microbial survival strategies under fluctuating environmental stressors and suggest that management practices interacting with seasonal cycles (e.g., manure application timing) may impact resistance dissemination [45,46]. While water samples were less consistently resistant, they showed intermittent spikes indicating their potential role as transient conduits in the spread of AMR within farm ecosystems.

The Multiple Antibiotic Resistance Index (MARI) values observed in this study highlight substantial resistance pressure within Tennessee small-scale farms. Elevated indices in *Enterococcus* indicate its role as a dominant reservoir, reflecting environmental persistence and likely exposure to selective pressures from antimicrobial use [47,48]. *E. coli* and *Klebsiella* also maintained indices above the 0.2 high risk threshold across several matrices and seasons, confirming that multiple genera contribute to environmental AMR risks. High MARI values in manure and soil are consistent with their recognition as key reservoirs shaped by practices such as manure application and grazing schedules [49]. Water samples generally showed lower indices, but occasional elevations, such as *Enterococcus* in fall water, indicate that hydrological pathways can serve as intermittent conduits of resistance dissemination [50].

The resistance pattern diversity analysis shows how resistance traits are structured within and across species. *Enterococcus* carried the most complex multidrug resistant signatures, spanning multiple drug classes [38,39]. *E. coli* and *Klebsiella* contributed fewer but ecologically relevant combinations, with peaks that often overlapped with those observed in *Enterococcus.* Shared resistance hubs, particularly AZM, ERY, AMP and VAN, suggest common selective pressures across matrices and are consistent with evidence that these antibiotics can act as resistance anchors in clinical and agricultural settings [51,52]. This cross-species convergence signals the potential for horizontal gene transfer and co selection, reinforcing concern about multidrug resistant reservoirs in small scale farming systems.

The BMP survey emphasizes the importance of farmer practices in shaping AMR dynamics on Tennessee farms. High engagement in veterinary consultation, antibiotic record keeping and BMP training suggests that many farmers are actively managing antimicrobial use, in line with reports that professional oversight can reduce misuse [53,54]. However, there remains room for improvement in documentation and especially in diversifying manure handling, which in this study was limited to stockpiling. It is encouraging that all respondents used formal dead animal disposal services because alternative practices such as burial or above ground disposal can allow resistant bacteria to enter soil and water, and similar links between suboptimal disposal and environmental contamination have been documented in other agricultural systems [55,56,57]. These good practices help limit the spread of resistance into key environmental reservoirs identified in this study: soil and manure functioned as primary sinks for resistance, while water can act as a transient conduit for dissemination [58,59]. Limited awareness of AMR as a public health threat among more than one third of surveyed farmers may further constrain adoption of improved practices. The overlap between BMP survey data and microbiological findings suggests that mitigation will require both educational and operational interventions, with priority given to manure management and strengthening farmer awareness of AMR risks. These findings show the need for regionally tailored stewardship programs in the southeastern U.S. that prioritize accessible manure management training, improved AMR literacy, and enhanced veterinary support. Extension services and state agencies could play a key role in promoting BMP adoption through farmer networks and incentive-based interventions.

This study has some limitations. First, we did not conduct genotypic analyses to confirm resistance genes, which may limit interpretation of resistance mechanisms. Second, the small number of farms and the cross-sectional seasonal design restrict generalizability and preclude longitudinal conclusions. Future studies should incorporate molecular confirmation and follow farms over time to better capture resistance trends.

## 4. Materials and Methods

### 4.1. Sample Collection

This study was conducted on 17 small-scale cattle farms in Middle Tennessee, USA, defined as operations with fewer than 200 cattle and primarily family-managed, in line with USDA guidelines. All animal-related procedures were reviewed and approved by the Tennessee State University Institutional Animal Care and Use Committee (IACUC; Animal Welfare Assurance #A4472-01). Sampling was performed quarterly to capture winter, spring, summer and fall conditions.

In total, 153 environmental samples were collected: soil (n = 63), manure (n = 54) and water (n = 36). Within each farm, sampling locations were selected to represent key management areas and reduce bias. Soil was collected from animal housing, manure storage and adjacent fields at 5–10 cm depth using sterile stainless-steel corers while avoiding areas with visible manure to minimize contamination from fecal matter. Approximately three subsamples (~200 g each) were pooled into a composite (~600 g), homogenized and cleared of visible debris. Manure samples (~500 g) were collected from three sites per farm using sterile scoops; portions were taken from the interior of piles less than two weeks old to minimize external contamination. Water (500 mL) was collected in sterile polypropylene bottles from troughs, ponds or streams by submerging bottles below the surface and sealing aseptically.

All samples were transported on ice (~4 °C) and processed within 24 h or stored briefly at 4 °C or −20 °C until analysis.

### 4.2. Bacterial Isolation and Identification

Soil and manure (25 g) obtained from the composite samples as described above were homogenized in 225 mL Buffered Peptone Water (BPW; Difco™ BD, Sparks, MD, USA) using a Stomacher^®^ 400 (Seward, Worthing, UK) for 1 min at medium speed. Homogenates were incubated for 24 h at 37 °C in nutrient broth (Difco™ BD, Sparks, MD, USA) (*E. coli*, *Klebsiella* spp.) or BBL Enterococcosel broth (BD BBL, Sparks, MD, USA) (*Enterococcus* spp.). Aliquots (10 µL) were streaked onto Eosin Methylene Blue (EMB) agar (Difco™ BD, Sparks, MD, USA) (*E. coli*), Klebsiella ChromoSelect agar (Sigma-Aldrich, St. Louis, MO, USA) (*Klebsiella* spp.) and Enterococcosel agar (BD BBL, Sparks, Sparks, MD, USA) (*Enterococcus* spp.) and incubated aerobically at 37 °C for 24–48 h.

Colonies with characteristic morphologies were purified and identified biochemically using API 20E (bioMérieux, Marcy-l’Étoile, France) for Gram negative isolates and API Strep (bioMérieux, Marcy-l’Étoile, France) for enterococci. Confirmed isolates were stored in 80 percent glycerol supplemented with tryptic soy broth (Difco™ BD, Sparks, MD, USA) at −80 °C until further analysis.

### 4.3. DNA Extraction and Molecular Confirmation

DNA was extracted from overnight cultures in tryptic soy broth using the UltraClean^®^ Microbial DNA Isolation Kit (MO BIO Laboratories, Carlsbad, CA, USA) according to the manufacturer’s instructions. PCR assays targeted the 16S rRNA gene (*E. coli*), *mdh* (*Klebsiella* spp.) and *tuf* (*Enterococcus* spp.) following published protocols [60,61,62]. Primer sequences, amplicon sizes and cycling conditions are summarized in Table 2.

PCR reactions were run on a ProFlex thermal cycler (Applied Biosystems, Carlsbad, CA, USA). Amplicons were resolved on 1–2% agarose gels (Sigma-Aldrich, St. Louis, MO, USA) stained with ethidium bromide (VWR International, Radnor, PA, USA) and visualized under UV illumination.

### 4.4. Antimicrobial Susceptibility Testing

Antimicrobial susceptibility was determined using the Kirby–Bauer disk diffusion method on Mueller–Hinton agar (Difco, BD, Sparks, MD, USA) in accordance with CLSI guidelines [63]. Overnight cultures were adjusted to 0.5 McFarland turbidity. Isolates were tested against 12 antibiotics: ERY (15 µg), NAL (30 µg), AMP (10 µg), MEM (10 µg), FEP (30 µg), VAN (30 µg), DOX (30 µg), CHL (30 µg), GEN (10 µg), IPM (10 µg), AZM (AZM, 15 µg) and CTX (30 µg). The antibiotics were selected to reflect a range of agents commonly used in both clinical and veterinary settings and to assess resistance trends in *Enterococcus*, *E. coli*, and *Klebsiella*. The panel included antibiotics relevant for monitoring resistance in these organisms, including those frequently associated with resistance in environmental and agricultural contexts. Inhibition zones were interpreted using CLSI breakpoints where available. For organism–antibiotic combinations lacking defined clinical breakpoints (e.g., VAN for *E. coli* and *Klebsiella*), zone diameter data were retained to allow cross-genus comparisons and detection of reduced susceptibility trends. Plates were incubated at 37 °C for 18–24 h, and inhibition zones were interpreted according to CLSI breakpoints. Appropriate ATCC reference strains such as *Enterococcus faecium* ATCC 19434 for *Enterococcus* spp., *Escherichia coli* ATCC 11775 for *E. coli* spp., and *Klebsiella pneumoniae* ATCC 700603 for *Klebsiella* spp. were included as quality control for each batch of tests. All antibiotic disks were obtained from Fisher Scientific Inc., Lenexa, KS, USA.

### 4.5. Multiple Antibiotic Resistance Index (MARI)

The MARI for each isolate was calculated using the formula *a/b*, where *a* is the number of antibiotics to which the isolate was resistant, and *b* is the total number of antibiotics tested. Isolates with MARI values greater than 0.2 were interpreted as originating from environments with frequent or intensive antibiotic exposure [64].

### 4.6. Farmer Survey on Management Practices

A structured questionnaire was administered to 26 farmers at the time of sample collection. The survey captured demographics, antibiotic use and record keeping, veterinary consultation, manure and carcass management, hygiene measures and awareness of antimicrobial resistance. Participation was voluntary, and written informed consent was obtained from all respondents.

### 4.7. Statistical Analysis

All statistical analyses were performed in R version 4.4.2 (R Core Team, Vienna, Austria). Descriptive statistics were used to summarize bacterial prevalence by matrix and season. Associations between detection, sample type and season were evaluated using chi square tests. Antimicrobial resistance data were visualized as heatmaps and violin plots using the ggplot2 and pheatmap packages. Comparisons of MARI values across seasons and sample types were conducted using nonparametric tests, with significance set at *p* < 0.05.

## 5. Conclusions

This study showed that small-scale cattle farms harbor commensal *Enterococcus* spp., *E. coli* and *Klebsiella* spp. with notable antimicrobial resistance. Manure and soil were the main reservoirs, with seasonal peaks in prevalence and multidrug resistance, while farm water occasionally carried resistant isolates. Frequent resistance to beta lactams, macrolides and glycopeptides suggests strong selection from commonly used antimicrobials, and MARI values above 0.2 for many manure and soil isolates indicate environments with substantial antibiotic exposure and potential for resistance maintenance.

The farmer survey indicated strong engagement with veterinarians and record keeping but revealed gaps in manure handling, carcass disposal and awareness of AMR risks. These findings show that environmental reservoirs and management practices act jointly to shape resistance patterns on small-scale farms. Strengthening manure and water management, improving carcass disposal, and integrating antimicrobial resistance awareness into existing extension activities represent practical next steps. Coupling these efforts with ongoing surveillance can help reduce resistance risks while supporting sustainable livestock production and protecting public health.

## Figures and Tables

**Table 1:** Demographics and farming characteristics in small-scale cattle farms in Tennessee.

Variable	Category	Frequencies	Percentages
Gender	Male	24	92.3
	Female	2	7.7
Age	35 and under	1	3.8
	36–54	2	7.7
	55–64	8	30.8
	65 and above	15	57.7
Education level	Bachelor’s Degree	4	15.4
	Associate degree	1	3.8
	Certificate	1	3.8
	GED	1	3.8
	High School Diploma	11	42.3
	Master’s Degree	6	23.1
	N/A	2	7.7
Livestock type	Beef	23	88.5
	Poultry	3	11.5
Number of animals	0–50	17	65.8
	51–99	6	22.8
	≥100	3	11.4
Antibiotics use	Disease prevention	1	3.8
	Treat sick animals only	18	69.2
	Treat sick animals/disease prevention	6	23.2
	Never	1	3.8
Maintain written records	Yes	15	57.7
	No	11	42.3
Veterinarians’ advice sought	Yes	20	76.9
	No	6	23.1
Antibiotic’s usage	As recommended	26	100
Awareness of AMR a public health threat	Yes	17	65.4
	No	9	34.6
Years raising livestock	0–20	13	50.0
	21–40	9	34.6
	≥60	4	15.4
Training on BMPs	Yes	22	84.6
	No	4	15.4
Extension agent consultation	Yes	26	100
Consultation frequency	Monthly	1	3.8
	Occasionally	2	7.7
	Often as recommended	1	3.8
	Only when needed	1	3.8
	Regularly	9	34.6
	Yearly	1	3.8
Dead animal disposal	Dead animal service	26	100
Fresh produce next to livestock	Yes	4	15.4
	No	22	84.6
Manure management practices	Stockpiling	26	100

**Table 2:** Primer sequences and target genes for *E. coli, Klebsiella,* and *Enterococcus* spp.

Target Bacteria	Primer Sequence (5′-3′)	Target Gene	Tm^2^ (°C) *	Amplified Segment (bp)	References
*E. coli*	FWD: 5′-GGTAACGTTTCTACCGCAGAGCTTG’3	16S rRNA	60.4	585	[60]
	REV: 5′-CAGGGTTGGTACACTGTCATTACG’3		60.2		
* Enterococcus * spp.	FWD: 5′-TACTGACAAACCATTCATGATG-3′	*tuf*	55.0	112	[61]
	REV: 5′AACTTCGTCACCAACGCGAAC-3′		56.5		
* Klebsiella * spp.	FWD: 5′-GCGTGGCGGTAGATCTAAGTCATA-3′	*mdh*	53	364	[62]
	REV: 5′ TTCAGCTTCGCCACAAAGGTA-3′				

* Melting temperature of primer.

## Data Availability

The original contributions presented in this study are included in the article. Further inquiries can be directed to the corresponding author.

## Supplementary Materials

The following supporting information can be downloaded at https://www.mdpi.com/article/10.3390/antibiotics15020217/s1, Table S1: Multiple Antibiotic Resistance (MAR) index values for *Enterococcus*, *Klebsiella*, and *Escherichia coli* from soil, water, and manure isolates in cattle farms in different seasons from Middle Tennessee.

## Abbreviations

The following abbreviations are used in this manuscript:

PCR	Polymerase chain reaction
MARI	Multiple Antibiotic Resistance Index
AMR	Antimicrobial resistance
CDC	Centers for Disease Control and Prevention
ESBL	Extended spectrum beta lactamase
USA	United States of America
ATCC	American Type Culture Collection
rRNA	Ribosomal ribonucleic acid
ERY	Erythromycin
AMP	Ampicillin
VAN	Vancomycin
GEN	Gentamicin
DOX	Doxycycline
CHL	Chloramphenicol
FEP	Cefepime
IPM	Imipenem
NAL	Nalidixic acid
CTX	Cefotaxime
MEM	Meropenem
AZM	Azithromycin
MDR	Multidrug resistance
BMP	Best management practice
GED	General Educational Development (high school equivalency credential)
IACUC	Institutional Animal Care and Use Committee
EMB	Eosin methylene blue (agar)
DNA	Deoxyribonucleic acid
Tm	Melting temperature
CLSI	Clinical and Laboratory Standards Institute
