# Supplementary material for: Susceptibility Profile and Multiple Antibiotics Resistance of Escherichia coli, Klebsiella spp., and Enterococci from Small-Scale Cattle Farms in Tennessee
Source: Antibiotics (Basel). 2026 Feb 17;15(2):217. doi: 10.3390/antibiotics15020217 (PMC12937398; doi:10.3390/antibiotics15020217)
Supplement: Supplementary file 1 [file antibiotics-15-00217-s001.zip › antibiotics-4099462-supplementary.pdf]

Supplementary Materials:

Table S1. Multiple Antibiotic Resistance (MAR) index values for *Enterococcus*, *Klebsiella*, and *Escherichia coli* from soil, water, and manure isolates in farms in different seasons from Middle Tennessee.

Season	Sample Type	Bacteria	Resistance to no. (%) agents [a]	Resistance Profile [b]	MAR index
Fall	Soil	*E. coli*	3(25) [a]	ERY-AMP-VAN	0.25
		Enterococcus	0(0.0) [a]	-	-
		Klebsiella	5(42) [ab]	ERY-NAL-AMP-VAN-AZM	0.42
	Manure	*E. coli*	4(33) [a]	ERY-AMP-VAN-AZM	0.33
		Enterococcus	5(42) [ab]	ERY-NAL-MEM-FEP-AZM	0.42
		Klebsiella	5(42) [ab]	ERY-AMP-FEP-VAN-AZM	0.42
	Water	*E. coli*	6(50) [ab]	ERY-AMP-VAN-DOX-AZM-CTX	0.50
		Enterococcus	7(58) [ab]	ERY-NAL-AMP-MEM-FEP-VAN-AZM	0.58
		Klebsiella	4(33) [a]	ERY-AMP-VAN-AZM	0.33
Spring	Soil	*E. coli*	9(75) [a]	ERY-AMP-MEM-FEP-VAN-CHL-DOX-AZM-CTX	0.75
		Enterococcus	11(92) [a]	ERY-NAL-AMP-MEM-FEP-VAN-IPM-CHL-DOX-AZM-CTX	0.92
		Klebsiella	6(50) [a]	ERY-NAL-AMP-VAN-AZM-CTX	0.50
	Manure	*E. coli*	5 (42) [a]	ERY-AMP-VAN-DOX-AZM	0.42
		Enterococcus	8 (67) [a]	ERY-NAL-AMP-FEP-VAN-CHL-DOX-CTX	0.67
		Klebsiella	6(50) [a]	ERY-NAL-AMP-VAN-DOX-CHL	0.50
	Water	*E. coli*	0(0) [b]	-	-

		Enterococcus	8(67) [a]	ERY-NAL-AMP-FEP-VAN-CHL-DOX-AZM	0.67
		Klebsiella	0(0) [bc]	-	-
Summer	Soil	*E. coli*	3(25) [a]	ERY-AMP-VAN	0.25
		Enterococcus	10(83) [b]	ERY-NAL-AMP-MEM-FEP-VAN-CHL-DOX-AZM-CTX	0.83
		Klebsiella	4(33) [a]	ERY-AMP-VAN-GEN	0.33
	Manure	*E. coli*	9(75) [bc]	ERY-NAL-AMP-VAN-CHL-GEN-DOX-AZM-CTX	0.75
		Enterococcus	11(92) [bd]	ERY-NAL-AMP-MEM-FEP-VAN-CHL-GEN-DOX-AZM-CTX	0.92
		Klebsiella	0(0)[a]	-	-
	Water	*E. coli*	6(50) [ab]	ERY-AMP-VAN-DOX-AZM-CTX	0.50
		Enterococcus	5(42) [ab]	NAL-MEM-FEP-VAN-DOX	0.42
		Klebsiella	7(58) [ab]	ERY-AMP-FEP-VAN-GEN-DOX-AZM	0.58
Winter	Soil	*E. coli*	8(67) [a]	ERY-AMP-FEP-VAN-CHL-DOX-AZM-CTX	0.67
		Enterococcus	8(67) [a]	ERY-NAL-MEM-FEP-VAN-CHL-AZM-CTX	0.67
		Klebsiella	0(0) [b]	-	-
	Manure	*E. coli*	8(67) [a]	ERY-NAL-AMP-VAN-CHL-DOX-AZM-CTX	0.67
		Enterococcus	9(75) [a]	ERY-NAL-MEM-FEP-VAN-GEN-DOX-AZM-CTX	0.75
		Klebsiella	0(0) [bc]	-	-
	Water	*E. coli*	5(42) [a]	ERY-NAL-AMP-VAN-DOX	0.42
		Enterococcus	0(0) [bd]	-	-
		Klebsiella	5(42) [a]	ERY-AMP-VAN-AZM-CTX	0.42

[a] Within this column, values with different letters A through C differ significantly ($p < 0.05$).

[b] ERY, erythromycin; NAL, nalidixic; AMP, ampicillin; MEM, meropenem; FEP, cefepime; VAN, vancomycin; DOX, doxycycline; CHL, chloramphenicol; GEN, gentamicin; IMP, imipenem; AZM, azithromycin; CTX, cefotaxime.
